# An integrated database-pipeline system for studying single nucleotide polymorphisms and diseases

**DOI:** 10.1186/1471-2105-9-S12-S19

**Published:** 2008-12-12

**Authors:** Jin Ok Yang, Sohyun Hwang, Jeongsu Oh, Jong Bhak, Tae-Kwon Sohn

**Affiliations:** 1 Korean BioInformation Center, Korea Research Institute of Bioscience and Biotechnology (KRIBB), Daejeon, 305-806, Korea; 2 Department of Bio and Brain Engineering, Korea Advanced Institute of Science and Technology (KAIST), Daejeon, Korea; 3 Department of Biochemistry, Yonsei University, Seoul, Korea

## Abstract

**Background:**

Studies on the relationship between disease and genetic variations such as single nucleotide polymorphisms (SNPs) are important. Genetic variations can cause disease by influencing important biological regulation processes. Despite the needs for analyzing SNP and disease correlation, most existing databases provide information only on functional variants at specific locations on the genome, or deal with only a few genes associated with disease. There is no combined resource to widely support gene-, SNP-, and disease-related information, and to capture relationships among such data. Therefore, we developed an integrated database-pipeline system for studying SNPs and diseases.

**Results:**

To implement the pipeline system for the integrated database, we first unified complicated and redundant disease terms and gene names using the Unified Medical Language System (UMLS) for classification and noun modification, and the HUGO Gene Nomenclature Committee (HGNC) and NCBI gene databases. Next, we collected and integrated representative databases for three categories of information. For genes and proteins, we examined the NCBI mRNA, UniProt, UCSC Table Track and MitoDat databases. For genetic variants we used the dbSNP, JSNP, ALFRED, and HGVbase databases. For disease, we employed OMIM, GAD, and HGMD databases. The database-pipeline system provides a disease thesaurus, including genes and SNPs associated with disease. The search results for these categories are available on the web page , and a genome browser is also available to highlight findings, as well as to permit the convenient review of potentially deleterious SNPs among genes strongly associated with specific diseases and clinical phenotypes.

**Conclusion:**

Our system is designed to capture the relationships between SNPs associated with disease and disease-causing genes. The integrated database-pipeline provides a list of candidate genes and SNP markers for evaluation in both epidemiological and molecular biological approaches to diseases-gene association studies. Furthermore, researchers then can decide semi-automatically the data set for association studies while considering the relationships between genetic variation and diseases. The database can also be economical for disease-association studies, as well as to facilitate an understanding of the processes which cause disease. Currently, the database contains 14,674 SNP records and 109,715 gene records associated with human diseases and it is updated at regular intervals.

## Background

Many researchers have studied the relationships between disease and biological variations such as single nucleotide polymorphisms (SNPs), copy number variation, sequence repeats and genetic rearrangement [1-3]. Recently, work on genetic (SNP) variation associated with diseases has become intense, as many genetic variations are thought to affect the structure and function of proteins, as a result of amino acid substitutions [4,5]. Significantly, SNPs, which report over 90% of genetic variation in the human genome [6], can have a major impact on how humans respond to disease, to drugs, and to other therapies. Therefore, SNP information is a great resource in biomedical studies, diagnostics, and drug development [7].

Many researchers studying disease associated SNPs require integrated information on SNPs and disease for two reasons. First, in order to capture relationships between SNPs and diseases, and then, to understand which genes cause disease and how that is impacted by SNPs. Second, disease-association researchers can save much time and effort in identifying the candidate disease-causing genes.

Despite the needs, existing servers contain insufficient information about SNP-disease associations. Because public databases for SNPs and diseases are large, complicated, and difficult to use, their integration is challenging. Therefore, we developed an integrated database-pipeline system for studying SNP and disease-association. We constructed a large database with comprehensive data on genes and SNPs associated with disease. In particular, the database-pipeline system allows biologists to retrieve integrated information on diseases, SNPs, and amino acid changes, along with functional annotation.

## Methods and results

**Figure 1:**
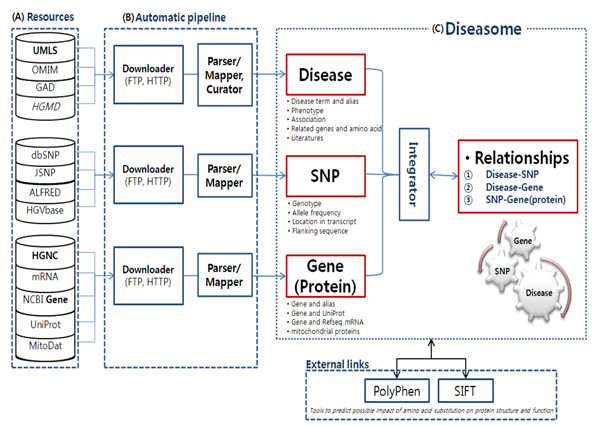
**Overview of the integrated database-pipeline system**. Rectangles represent computational applications, and are three in number. The Resource (A) contains gene-, SNP-, and disease-related primary resources and constructs a primary information database. The Automatic pipeline (B) retrieves information from primary databases and extracts essential gene-, SNP-, and disease-related data. We mapped disease terms and aliases, or gene names and aliases, based on the UMLS and HGNC databases. Also, disease terms were corrected for noun modification, stop word, and suffix. SNP effects were investigated by amino acid substitution; locations are available. The Diseasome (C) is a database including three categories of information (gene, SNP, and disease), and relationships among the three categories.

The integrated database-pipeline system of genes and SNPs associated with diseases was developed in three parts as shown in Figure 1. By using this pipeline system, we downloaded and extracted primary information from 13 public and private databases. Next, we unified complex disease terms and gene names, and constructed an integrated database which contains the three sub-categories of diseases; genes and proteins; and SNPs.

### Automatic collection and update of public resources

The integrated database-pipeline system uses file transfer protocol, hypertext transfer protocol, and JAVA-based data-extracting modules. The system also has a support function to design the database schema and to create the modules based on a graphic user interface. The integration pipeline system checks the updated data and downloads such data automatically from 13 public and private resource servers, and then informs the system administrator by e-mail. We selected the following representative databases for the disease, SNP, and gene resources: The disease category is updated from the databases Unified Medical Language System (UMLS) [8], Online Mendelian Inheritance in Man (OMIM) [9], Gene Association Database (GAD) [10], and Human Gene Mutation Database (HGMD) [11]. The gene and protein category is updated from the databases NCBI [12], HUGO Gene Nomenclature Committee (HGNC) [13], UniProt ([14], UCSC[15], and MitoDat (Mendelian Inheritance and the Mitochondrion) [16]. The genetic variation category (SNPs) is updated from the databases dbSNP [17], JSNP [18], ALFRED (Allele Frequency Database) [19], and HGVbase (Human Genome Variation database) [20]. The system is updated regularly, as the pipeline aquires data automatically.

### Defining disease terms based on the UMLS

The disease terms commonly used in many research articles and several disease databases such as OMIM, GAD, and HGMD have the character of natural language; there are many synonyms and slightly different expressions which refer to the same concept. We required a unified controlled vocabulary of disease names and their synonyms, to construct a non-redundant disease database. We accomplished this by using UMLS (Release Archive 2007AA), which is a very large, multi-purpose vocabulary database containing information about biomedical and health-related concepts, the various terms used, and the relationships among them. Moreover, UMLS successfully integrates widely used clinical terms, in sub-bases such as "Systematized Nomenclature of Medicine – Clinical Terms and Medical Subject Headings," so UMLS was an excellent resource allowing us to relate our database terms to medical informatics.

In addition, disease terms are associated with various word formations (in particular, noun modification). To solve this text stemmer problem, we defined disease terms expressed in public disease databases using four steps. First, we removed stop words employing a stop word list provided by OMIM. Second, we removed suffixes such as ", -es and -s. Third, we removed typographical errors and special characters. Finally, we mapped these processed disease terms to unique clinical concepts by comparing the terms with their several synonyms and different expressions as provided by UMLS [8].

### Defining genes according to HGNC and NCBI data

To permit the exploration of SNP effects on genetic variation, we adopted various gene annotations including gene Information from NCBI, RefSeq mRNA of UCSC Table Track, protein information from UniProt, and the mitochondrial biogenesis and function criteria of MitoDat (Mendelian Inheritance and the Mitochondrion). Because the mitochondrion has a central role in cellular metabolism, the mitochondrion is involved in many human diseases [16]. We integrated gene and protein data into the SNP and diseases resources based on a gene-synonym table from HGNC and gene information at NCBI. Next, we mapped UniProt proteins onto NCBI genes by BLAST search. Finally, we added mitochondrial gene and protein data from MitoDat, because this database predominantly contains information on human nuclear-encoded mitochondrial proteins.

### Integration of genes, SNPs, and diseases

To construct SNP-related information, we collected representative genetic variation (SNP), resources from dbSNP, JSNP, ALFRED, HGVbase, POLYPHEN (Polymorphism Phenotyping) [21], and SIFT (Sorting Intolerant From Tolerant) [22]. Finally, we integrated the information to show the interrelationships among SNPs located in genes, genes associated with diseases, and SNPs associated with diseases. The HGVbase database was adapted to integrate a curated resource describing human DNA variation and phenotype relationships. To predict a possible SNP impact of amino acid substitution on protein structure and function, we also linked to PolyPhen and SIFT. ALFRED contains data on allele frequencies at particular SNP loci for diverse populations, with reference to SNPs in dbSNP and JSNP. Our system can update primary databases automatically in real time. However, to integrate the various databases, we need to note the variations and partially update manually.

Next, we analyzed the influence of SNP location (e.g., CDS, UTR, Intron, or Promoter) on gene structure, and the effects of synonymous or non-synonymous SNPs on genetic variation and genes associated with disease, employing BLAST [23]. We explored amino acid changes caused by codon changes, and identified the locations of the altered amino acids in proteins. In addition, we determined whether SNPs were synonymous or non-synonymous, and identified the relationships of SNPs to candidate disease-causing genes.

## Web interface

**Figure 2:**
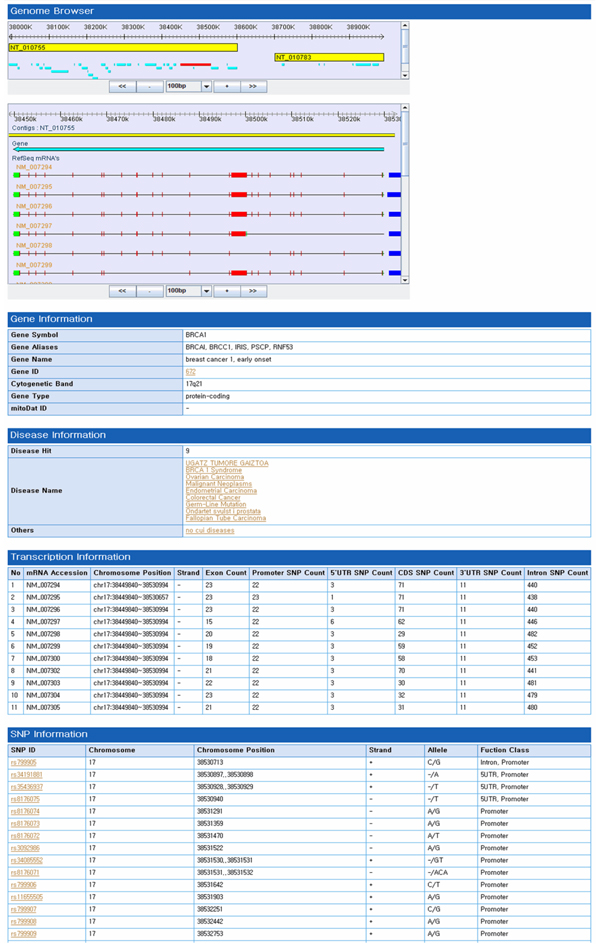
**Query table results and graphic viewer**. The retrieval page of the integrated gene, SNP, and diseases database. The information on diseases, genes, and SNP markers found as result of a query (e.g., BRCA1) are shown. When a user queries a gene symbol, the system retrieves the Gene Information table, which shows various gene annotations, disease information related to the queried gene, transcript information including the number of SNPs located in each transcript, and SNP information associated with the queried gene. In addition, the user can explore the data on gene-related transcripts, SNPs, and disease information, using the genome browser. If a user requires more specific information on any item, the user can click on a disease term, a gene ID, or a genetic variation number (SNP rs number).

The database server was implemented in JAVA and Java Server Pages connected to MySQL. The main web interface provides two ways to explore integrated disease-related information through a tree view of disease terms and through query searching. Users can look over all the disease terms together in the tree view. When users click on a disease term, they can obtain results consisting of targeted disease information (disease name, synonyms, and title), gene information, and SNP information directly. The web interface also allows querying with three kinds of terms: (1) a SNP identifier (rs number from dbSNP), (2) a gene ID (symbol and description), or, (3) a disease term. When the user submits a gene, the system will return a list of genes related to the query along with gene aliases and types. When selecting a specific gene, query results show the gene, gene transcripts, SNPs, and genetic variants through the genome browser [24]. The genome browser provides insights into the effects of genetic variations on transcripts. Transcripts and SNP marker information displayed in the genome browser facilitates the recognition of characteristics of disease-causing genes, especially if the SNP or genetic variation lies in the promoter region or in an intronic sequence [25]. A display of the gene, transcripts, SNPs, and disease information is shown in Figure 2.

To show a disease search, we present query results for diabetes. The results from the integrated disease- and genetic variation-related databases are more helpful to researchers than results from one database only. It provides more comprehensive information on the genes and SNP markers associated with the disease. For example, when using only one disease-related database for diabetes, researchers can obtain either disease-association study information from GAD or information on disease-related literatures from OMIM. Conversely, when using the integrated database-pipeline system, we obtain a list of genes associated with diabetes, and an SNP marker (rs1805097) associated with diabetes by making both the integrated disease and the genetic variation information available simultaneously. This integrated information allows researchers to consider the SNP effects on the gene along with relationships between SNPs and disease. The SNP marker (rs1805097) is located in the human insulin receptor substrate-2 (IRS-2) gene, which is a primary progesterone response gene. This SNP can affect amino acid change (GLY1057ASP), which has the possible impact of an amino acid substitution on the structure and function of a human protein [26]. Because this also includes the genome locations of disease-associated genes, effects of non-synonymous SNPs at the protein level, and disease-causing risk scores, users can expect to have a better understanding of the molecular causes of the disease.

## Conclusion and future direction

We constructed the integrated database for the study of genetic variation in disease, using an automatic integration pipeline system. Specifically, the database contains information on 124,389 disease, 12,445,925 SNP markers, and 38,597 genes, and includes 14,674 SNP records and 109,715 gene records associated with human diseases. A total of 1,319 SNPs cause amino acid changes, inevitably leading to severe disruptions of protein structure or function.

Consequently, the integrated database-pipeline system can be an indispensable resource. The system can economically facilitate disease-association studies by identifying candidate genes associated with disease, and genetic variation. It can aid the understanding of the genes which cause diseases and the impact of SNPs on diseases, by showing the relationships among genes, SNPs and diseases. The tool uses unified disease terms, which facilitates the outreach and extension of this database to various other medical sources. As the resources in this database-pipeline system are expanding continuously, we are planning to collect validated resources used in the detection of genetic variation for comparative studies.

## Competing interests

The authors declare that they have no competing interests.

## Authors' contributions

JOY wrote the manuscript and helped to update the website. SH also wrote the manuscript and designed the databases. JSO developed the website, and updates the system. JB directed this project and helped to draft the manuscript. TKS designed the database and helped to draft the manuscript. All authors read and approved the final manuscript.
